# Prognostic and clinicopathological impacts of Controlling Nutritional Status (CONUT) score on patients with gynecological cancer: a meta-analysis

**DOI:** 10.1186/s12937-023-00863-8

**Published:** 2023-07-08

**Authors:** Zheng Niu, Bing Yan

**Affiliations:** 1 Department of Gynecology, Affiliated Hangzhou First People’s Hospital, Zhejiang University School of Medicine, Hangzhou, Zhejiang China; 2 Department of Pharmacy, The First Affiliated Hospital of Huzhou University, Huzhou, Zhejiang China

**Keywords:** CONUT, Meta-analysis, Gynecological cancer, Prognosis, Evidence-based medicine

## Abstract

**Background:**

The Controlling Nutritional Status (CONUT) score has proven to be a potential biomarker for determining the prognosis of patients with various types of cancer. Its value in determining the prognosis of patients with gynecological cancer, however, remains unknown. The present study was a meta-analysis that aimed to evaluate the prognostic and clinicopathological significance of the CONUT score in gynecological cancer.

**Methods:**

The Embase, PubMed, Cochrane Library, Web of Science, and China National Knowledge Infrastructure databases were comprehensively searched through November 22, 2022. A pooled hazard ratio (HR), together with a 95% confidence interval (CI), was used to determine whether the CONUT score had prognostic value in terms of survival outcomes. Using odds ratios (ORs) and 95% CIs, we estimated the relationship between the CONUT score and clinicopathological characteristics of gynecological cancer.

**Results:**

We evaluated 6 articles, involving a total of 2,569 cases, in the present study. According to the results of our analyses, higher CONUT scores were significantly correlated with decreased overall survival (OS) (*n* = 6; HR = 1.52; 95% CI = 1.13–2.04; *P* = 0.006; I2 = 57.4%; Ph = 0.038) and progression-free survival (PFS) (*n* = 4; HR = 1.51; 95% CI = 1.25–1.84; *P* < 0.001; I2 = 0; Ph = 0.682) in gynecological cancer. Moreover, higher CONUT scores were significantly correlated with a histological grade of G3 (*n* = 3; OR = 1.76; 95% CI = 1.18–2.62; *P* = 0.006; I2 = 0; Ph = 0.980), a tumor size ≥ 4 cm (*n* = 2; OR = 1.50; 95% CI = 1.12–2.01; *P* = 0.007; I2 = 0; Ph = 0.721), and an advanced International Federation of Gynecology and Obstetrics (FIGO) stage (*n* = 2; OR = 2.52; 95% CI = 1.54–4.11; *P* < 0.001; I2 = 45.5%; Ph = 0.175). The correlation between the CONUT score and lymph node metastasis, however, was not significant.

**Conclusions:**

Higher CONUT scores were significantly correlated with decreased OS and PFS in gynecological cancer. The CONUT score, therefore, is a promising and cost-effective biomarker for predicting survival outcomes in gynecological cancer.

**Supplementary Information:**

The online version contains supplementary material available at 10.1186/s12937-023-00863-8.

## Introduction

Gynecological cancer comprises a series of heterogeneous cancers, predominantly cervical cancer (CC), endometrial cancer (EC), and ovarian cancer (OC) [1]. Globally, gynecological cancer is a serious public health issue, and according to the Global Cancer Incidence, Mortality, and Prevalence (GLOBOCAN) estimates, there were 1,398,601 gynecological cancer cases diagnosed and 671,875 associated deaths worldwide in 2020 [2]. The standard treatment methods for gynecological cancer include surgical resection, chemotherapy, radiotherapy, and immunotherapy with immune checkpoint inhibitors [3, 4]. Anticancer therapies, however, generally lead to a variety of side effects, which may compromise survival benefits. Effective prognostic biomarkers are pivotal for ensuring that precision medicine is provided to individual patients, which in turn improves the survival outcomes of patients with gynecological cancer.

Prognoses of gynecological cancer patients can be made using many novel inflammatory markers found in peripheral blood [5, 6]. These nutritional and inflammatory indices, including the systemic immune-inflammation index [5], C-reactive protein/albumin ratio [7], modified Glasgow Prognostic Score [8], prognostic nutritional index [9], and Controlling Nutritional status (CONUT) score [10], are easily accessible and cost-effective to utilize. The CONUT score is an evaluation of nutrition based on the serum albumin (ALB) level, lymphocyte count, and cholesterol level and was first proposed by Ignacio et al. as a routine assessment with which to evaluate the nutritional status of all inpatients [11]. The scoring system used to calculate the CONUT score is presented in Table 1 [ 11]. Increased CONUT scores are typically associated with an unfavorable nutritional status and weakened immune responses. Numerous studies have explored whether the CONUT score can be used to predict gynecological cancer survival; however, no consistent outcomes have been obtained [10, 12–16]. An increased CONUT score has been suggested, in certain articles, to be significantly related to a poor prognosis in gynecological cancer [10, 13, 15]. Other studies, however, have demonstrated no obvious relationship between the CONUT score and survival outcomes in cases of gynecological cancer [12, 16]. Therefore, in the present study, we performed a comprehensive literature search and conducted a meta-analysis to identify the relation of the CONUT score with the prognosis and clinicopathological characteristics of patients with gynecological cancer.

**Table 1:** The scoring system of CONUT score

Parameter	CONUT			
	Normal	Light	Moderate	Severe
Serum albumin (g/dL)	3.5–4.5	3.0–3.49	2.5–2.99	< 2.5
Score	0	2	4	6
Total lymphocytes (count/mm^3^)	≥ 1600	1200–1599	800–1199	< 800
Score	0	1	2	3
Cholesterol (mg/dl)	> 180	140–180	100–139	< 100
Score	0	1	2	3
Total CONUT score	0–1	2–4	5–8	9–12

*CONUT* controlled nutritional status score

## Materials and methods

### Study guidelines

The present meta-analysis was performed following the Preferred Reporting Items for Systematic Reviews and Meta-Analyses (PRISMA) guidelines [17].

### Literature retrieval

The PubMed, Embase, Web of Science, Cochrane Library, and China National Knowledge Infrastructure databases were systematically searched through November 22, 2022, using the following terms: (“Controlling Nutritional Status” or “CONUT”) AND (“endometrial neoplasm” OR “endometrial carcinoma” OR “endometrial cancer” OR “gynecological cancer” OR “gynecological carcinoma” OR “cervical cancer” OR “cervical carcinoma” OR “ovarian cancer” OR “ovarian carcinoma” OR “vulvar cancer” OR “vaginal cancer”). Studies published in all languages were eligible for inclusion, and we manually checked the reference lists of relevant studies to identify additional potentially eligible studies.

### Selection standards

We utilized the Population-Intervention-Control-Outcome-Study (PICOS) framework to develop the inclusion criteria for the present study [18], as follows: (1) population, patients with gynecological cancer based on a pathological or histological diagnosis; (2) intervention – exposure, pretreatment serum ALB, total cholesterol (TC), and total lymphocyte count were obtained to calculate the CONUT score and identify patients with a high score; (3) control, patients with a low pretreatment CONUT score and a normal nutritional status; (4) outcomes, studies published in any language evaluating the relationship between the CONUT score and survival in gynecological cancer, with available hazard ratios (HRs) and 95% confidence intervals (CIs) related to patient survival; and (5) study design, retrospective or prospective studies published in any language. For the intervention and control, a cutoff value was determined and used to divide patients into low and high CONUT score groups.

The exclusion criteria were as follows: (1) reviews, case reports, conference abstracts, letters, and comments; (2) articles that did not include sufficient information to analyze patient survival; (3) articles in which a cutoff value was not determined; and (4) nonhuman studies.

### Data collection and quality evaluation

Qualified articles were evaluated by two reviewers (ZN and BY), and any disagreement was resolved by reaching a consensus. The following data were collected for each eligible study: first author, year of publication, study country, sample size, cancer type, age, study duration, International Federation of Gynecology and Obstetrics (FIGO) stage, treatment, study center, follow-up, CONUT cutoff value, survival endpoints, study design, HR analysis type, adjustment covariates, HRs, and 95% CIs. If survival odds were determined through both univariate and multivariate analyses, the HRs and 95% CIs obtained through multivariate regression were used. The primary outcome of the present meta-analysis was the prognostic value of the CONUT score in regard to overall survival (OS) and progression-free survival (PFS) in patients with gynecological cancer. The secondary outcomes were the relationships between the CONUT score and the clinicopathological features of patients with gynecological cancer. Study quality was independently evaluated by two reviewers (ZN, BY) using the Newcastle–Ottawa Scale (NOS) [19], for which the maximum score was 9. Articles with NOS scores ≥ 6 were regarded as high-quality studies.

### Statistical analysis

We used combined HRs and 95% CIs to evaluate whether the CONUT score could be used to determine the prognosis of patients with gynecological cancer. The chi-square test and I^2^ statistic were utilized to estimate interstudy heterogeneities. When *P* < 0.10 and I^2^ > 50%, which indicated the presence of obvious heterogeneity among studies, we utilized a random-effects model; otherwise, we utilized a fixed-effects model. We then performed subgroup analyses, which were stratified by country, sample size, cancer type, FIGO stage, treatment, study center, age, NOS score, and cutoff value. We also conducted a leave-one-out sensitivity analysis to test whether our overall findings were robust. The relationship between the CONUT score and the clinicopathological features of patients with gynecological cancer was estimated by combining ORs and 95% CIs. Possible publication bias was evaluated using a funnel plot, Egger’s test, and Begg’s test. Statistical analysis was performed using Stata version 12.0 (Stata Corporation, College Station, TX, USA), in which *P* < 0.05 was considered to indicate a statistically significant difference.

### Ethnics statement

Patient consent and ethics approval were not required for the present study because all data were extracted from previously published literature.

## Results

### Eligible studies

**Fig. 1:**
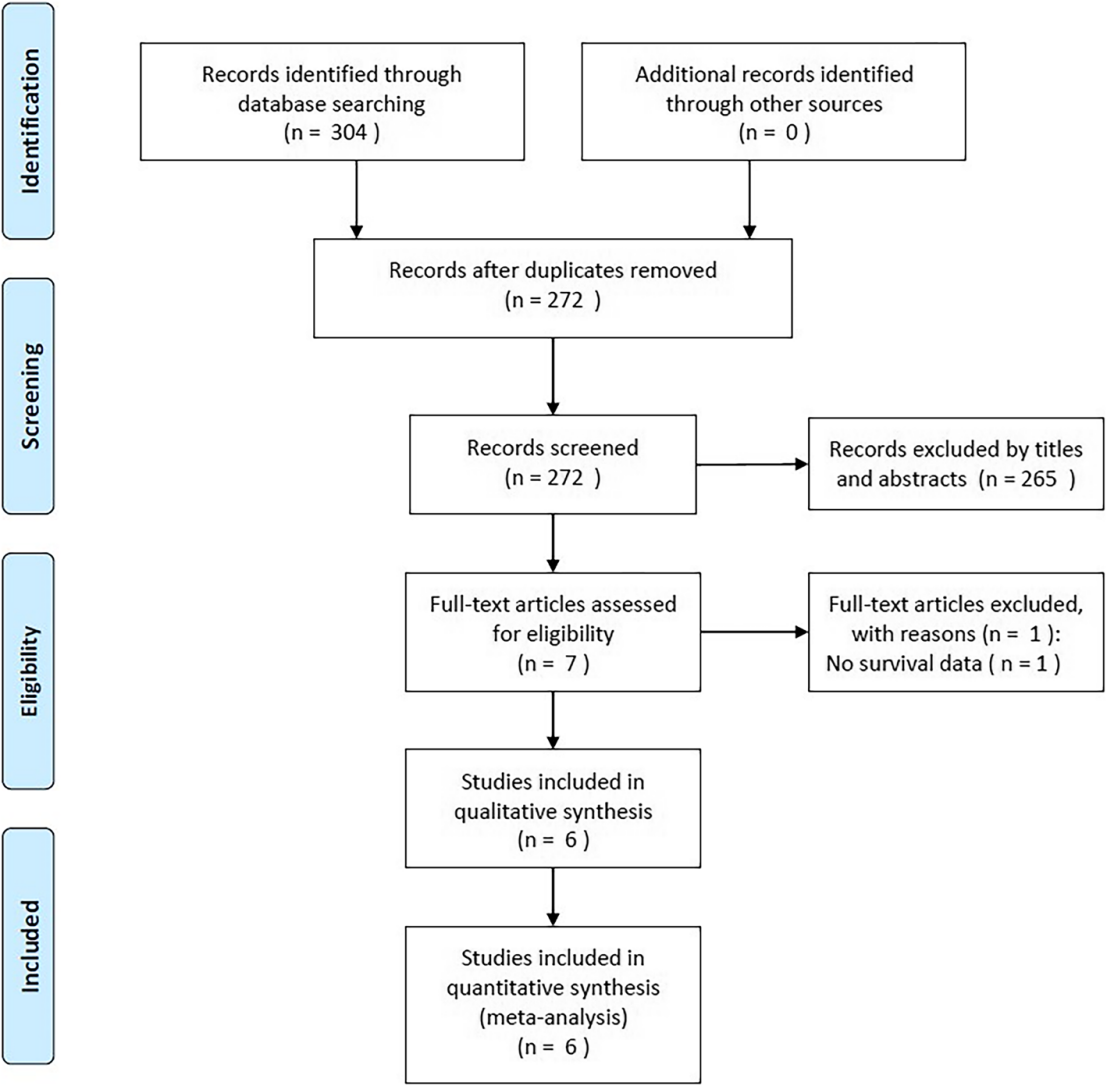
Study selection flowchart according to the Preferred Reporting Items for Systematic Reviews and Meta-Analysis guidelines

Figure 1 shows the study selection flowchart. The initial search identified 304 studies, 272 of which remained after duplicates were removed. Through a review of the titles and abstracts, 265 additional studies were excluded, leaving 7 articles for further evaluation via full-text examination. One additional study was excluded because it did not contain survival data for analysis. In total, the present meta-analysis enrolled 6 studies, encompassing 2,569 patients [10, 12–16] (Fig. 1; Table 2).

**Table 2:** Basic characteristics of included studies in this meta-analysis

Study	Year	Country	Sample size	Cancer type	Study period	Age (year) Median (range)	FIGO stage	Treatment	Study center	Follow-up (month) Median (range)	Endpoint	Cut-off value	HR analysis	Adjustment covariates for multivariate analysis	NOS score
Li, Y [10]	2020	China	206	Ovarian cancer	2013–2016	58	I-IV	Surgery	Single center	1–80	OS, PFS	3	Multivariate	Age, menopause, FIGO stage, histological subtype, histological grade, ascites, CA125, treatment, residual disease, chemosensitivity	7
Li, Q [12]	2021	China	1,038	Endometrial cancer	2012–2017	56 (51–61)	I-IV	Surgery	Single center	1–84	OS, PFS	1	Multivariate	TC, NLR, CA125, NPS, age, menopause, histological subtype, histological grade, FIGO stage, myometrial invasion, treatment	8
Zhang, G [13]	2021	China	698	Cervical cancer	2004–2015	51	I-II	Surgery + CCRT	Multicenter	56.2 (4.9–186.9)	OS, PFS	3	Multivariate	Age, BMI, FIGO stage, hemoglobin, histological type, lymph nodes metastasis, parametrial invasion, positive resection margin, SCC-Ag, PNI, HDR brachytherapy	7
Bekos, C [14]	2022	Austria	337	Ovarian cancer	2000–2015	59.54	I-IV	Surgery + chemotherapy	Single center	1–120	OS, PFS	2	Multivariate	FIGO stage, age, histological grade, residual disease, histological type	7
Jiang, L [15]	2022	China	122	Cervical cancer	2016–2018	48.3 (32–67)	I-II	Surgery	Single center	1–60	OS	3	Multivariate	Age, histological type, histological grade, positive resection margin, SCC-Ag, tumor size, lymph nodes metastasis, FIGO stage, parametrial invasion, treatment	7
Karakaş, S [16]	2022	Turkey	168	Ovarian cancer	2015–2020	55.7	I-IV	Surgery	Single center	26.3	OS	1.5	Multivariate	Menopause, FIGO stage, ascites, PNI	8

*FIGO* International Federation of Gynecology and Obstetrics, *CCRT* concurrent chemoradiotherapy, *OS* overall survival, *PFS* progression-free survival, *HR* hazard ratio, *NOS* Newcastle-Ottawa Scale, *TC* total cholesterol, *NLR* neutrophil to lymphocyte ratio, *NPS* Naples prognostic score, *BMI* body mass index, *SCC-Ag* squamous cell carcinoma antigen, *PNI* prognostic nutritional index

### Enrolled article features

Table 2 displays the features of the articles enrolled in the present meta-analysis. The year of publication for the included articles ranged from 2020 to 2022. Four articles were conducted in China [10, 12, 13, 15], one in Austria [14], and one in Turkey [16], all of which were retrospective in nature. Five articles were published in English [10, 12–14, 16] and one in Chinese [15]. Three studies included patients with OC [10, 14, 16], two included those with CC [13, 15], and one included those with EC [12]. The present meta-analysis included articles with study populations of 206–1,038 (median, 252.5). Four articles included patients with FIGO stage I–IV cancer [10, 12, 14, 16] while two included patients with FIGO stage I–II cancer [13, 15]. Five studies were single-center studies [10, 12, 14–16], and one was a multicenter study [13]. Three studies utilized a threshold of 3 for the CONUT score [10, 13, 15], while one each utilized a threshold of 1 [12], 2 [14], and 1.5 [16]. Six articles mentioned the value of the CONUT score in predicting OS [10, 12–16], while four mentioned the relationship of the CONUT score with PFS [10, 12–14]. Multivariate regression was conducted to extract HRs with their associated 95% CIs. The median NOS score was 7 (range, 7–8), indicating the high quality of the articles included in the present meta-analysis (Table 2).

### Value of the CONUT score in predicting OS

**Fig. 2:**
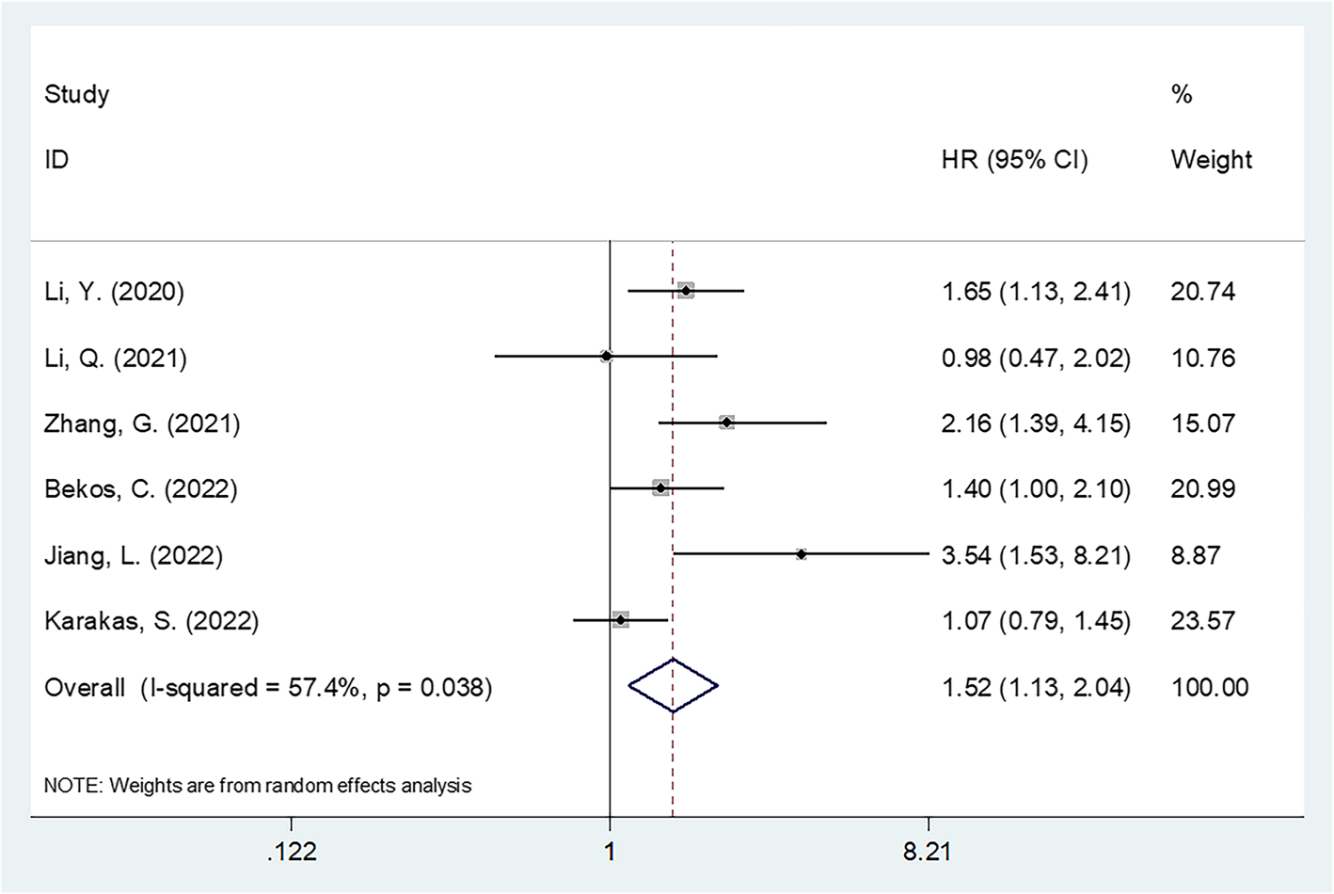
Forest plot of the association between CONUT and overall survival in gynecological cancer

The 6 articles enrolled in the present study included 2,569 cases of gynecological cancer [10, 12–16] and showed that the CONUT score could be used to predict OS. Obvious heterogeneity was detected among the studies included in the present meta-analysis; therefore, we applied a random-effects model (I^2^ = 57.4%, *P* = 0.038). The combined data obtained (HR = 1.52, 95% CI = 1.13–2.04, *P* = 0.006) suggested that an increased CONUT score had a significant relationship with decreased OS in patients with gynecological cancer (Fig. 2; Table 3). As revealed by subgroup analyses, increased CONUT scores were significantly related to poor OS regardless of the FIGO stage or study center (Table 3). Furthermore, based on subgroup analyses, an increased CONUT score was significantly related to poor OS under the following conditions: studies conducted in China (*P* < 0.001); sample size ≥ 300 (*P* = 0.006); OC (*P* = 0.009); and CC (*P* < 0.001); treatment with surgery + concurrent chemoradiotherapy (CCRT) (*P* = 0.003); median patient age ≥ 56 years (*P* = 0.004); and a CONUT score threshold of 3 (*P* < 0.001) (Table 3).

**Table 3:** Subgroup analysis of the association between CONUT and overall survival in patients with gynecological cancer

Subgroups	No. of studies	No. of patients	Effects model	HR (95% CI)	p	Heterogeneity	
						I^2^(%)	Ph
Total	6	2,569	Random	1.52 (1.13–2.04)	0.006	57.4	0.038
Country							
China	4	2,064	Fixed	1.77 (1.35–2.33)	< 0.001	48.4	0.121
Others	2	505	Fixed	1.19 (0.94–1.51)	0.143	17.2	0.272
Sample size							
< 300	3	496	Random	1.63 (0.96–2.78)	0.071	76.1	0.015
≥ 300	3	2,073	Fixed	1.49 (1.12–1.98)	0.006	36.6	0.207
Cancer type							
Ovarian cancer	3	711	Fixed	1.31 (1.07–1.59)	0.009	38.7	0.196
Cervical cancer	2	820	Fixed	2.50 (1.58–3.96)	< 0.001	0	0.334
Endometrial cancer	1	1,038	-	0.98 (0.47–2.03)	0.948	-	-
FIGO stage							
I-IV	4	1,749	Fixed	1.28 (1.06–1.55)	0.012	21.6	0.281
I-II	2	820	Fixed	2.50 (1.58–3.96)	< 0.001	0	0.334
Treatment							
Surgery	4	1,534	Random	1.46 (0.94–2.26)	0.089	67.2	0.028
Surgery + CCRT/ Surgery + chemotherapy	2	1,035	Fixed	1.60 (1.18–2.18)	0.003	39.2	0.200
Study center							
Single center	5	1,871	Random	1.42 (1.04–1.94)	0.027	56.5	0.056
Multicenter	1	698	-	2.16 (1.25–3.74)	0.006	-	-
Cut-off value							
= 3	3	1,026	Fixed	1.95 (1.46–2.61)	< 0.001	29.1	0.244
≠ 3	3	1,543	Fixed	1.17 (0.94–1.46)	0.170	0	0.480
Age (median), years							
< 56	3	988	Random	1.86 (0.93–3.73)	0.080	80.2	0.006
≥ 56	3	1,581	Fixed	1.44 (1.12–1.85)	0.004	0	0.447
NOS score							
< 8	4	1,363	Fixed	1.72 (1.37–2.16)	< 0.001	36.6	0.193
≥ 8	2	1,206	Fixed	1.06 (0.80–1.40)	0.705	0	0.820

*CONUT* controlled nutritional status score, *FIGO* International Federation of Gynecology and Obstetrics, *CCRT* concurrent chemoradiotherapy, *NOS* Newcastle–Ottawa Scale

### Significance of the CONUT score in predicting PFS

**Fig. 3:**
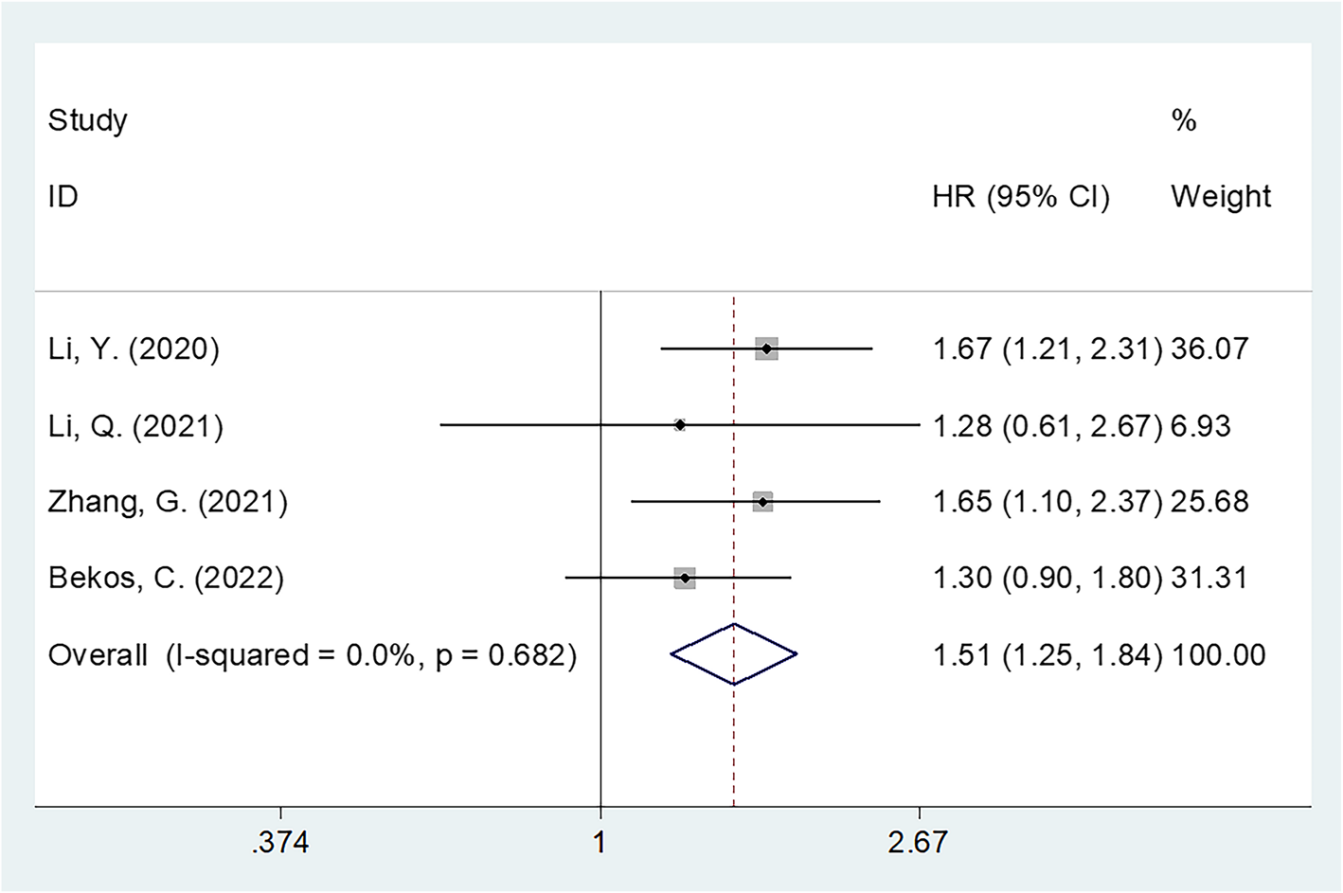
Forest plot of the association between CONUT and progression-free survival in gynecological cancer

Of the articles included for analysis in the present study, a total of 4 studies, involving 2,279 patients [10, 12–14], presented data on the relationship between the CONUT score and PFS in patients with gynecological cancer. There was low heterogeneity (I^2^ = 0%, *P* = 0.682); therefore, we utilized a fixed-effects model (Fig. 3; Table 4). Based on the pooled results, increased CONUT scores were significantly correlated with decreased PFS in patients with gynecological cancer (HR = 1.51; 95% CI = 1.25–1.84; *P* < 0.001) (Fig. 3; Table 4). As shown in Table 4, based on subgroup analyses, an increased CONUT score was significantly correlated with poor PFS regardless of sample size, FIGO stage, treatment, age, or study center (all *P* < 0.05).

**Table 4:** Subgroup analysis of the association between CONUT and progression-free survival in patients with gynecological cancer

Subgroups	No. of studies	No. of patients	Effects model	HR (95% CI)	p	Heterogeneity	
						I^2^(%)	Ph
Total	4	2,279	Fixed	1.51 (1.25–1.84)	< 0.001	0	0.682
Country							
China	3	1,942	Fixed	1.62 (1.28–2.05)	< 0.001	0	0.803
Others	1	337	-	1.30 (0.92–1.84)	0.138	-	-
Sample size							
< 300	1	206	-	1.67 (1.21–2.31)	0.002	-	-
≥ 300	3	2,073	Fixed	1.43 (1.12–1.82)	0.004	0	0.630
Cancer type							
Ovarian cancer	2	543	Fixed	1.49 (1.17–1.88)	0.001	7.3	0.299
Cervical cancer	1	698	-	1.65 (1.13–2.42)	0.010	-	-
Endometrial cancer	1	1,038	-	1.28 (0.61–2.67)	0.511	-	-
FIGO stage							
I-IV	3	1,581	Fixed	1.47 (1.17–1.84)	0.001	0	0.542
I-II	1	698	-	1.65 (1.13–2.42)	0.010	-	-
Treatment							
Surgery	2	1,244	Fixed	1.60 (1.19–2.15)	0.002	0	0.516
Surgery + CCRT/ Surgery + chemotherapy	2	1,035	Fixed	1.45 (1.12–1.87)	0.005	0	0.363
Study center							
Single center	3	1,581	Fixed	1.47 (1.17–1.84)	0.001	0	0.542
Multicenter	1	698	-	1.65 (1.13–2.42)	0.010	-	-
Cut-off value							
= 3	2	904	Fixed	1.66 (1.30–2.13)	< 0.001	0	0.964
≠ 3	2	1,375	Fixed	1.30 (0.95–1.77)	0.105	0	0.970
Age (median), years							
< 56	1	698	-	1.65 (1.13–2.42)	0.010	-	-
≥ 56	3	1,581	Fixed	1.47 (1.17–1.84)	0.001	0	0.542
NOS score							
< 8	3	1,241	Fixed	1.53 (1.25–1.87)	< 0.001	0	0.525
≥ 8	1	1,038	-	1.28 (0.61–2.67)	0.511	-	-

*CONUT* controlled nutritional status score, *FIGO* International Federation of Gynecology and Obstetrics, *CCRT* concurrent chemoradiotherapy, *NOS* Newcastle–Ottawa Scale

### Association of the CONUT score with clinicopathological characteristics

**Fig. 4:**
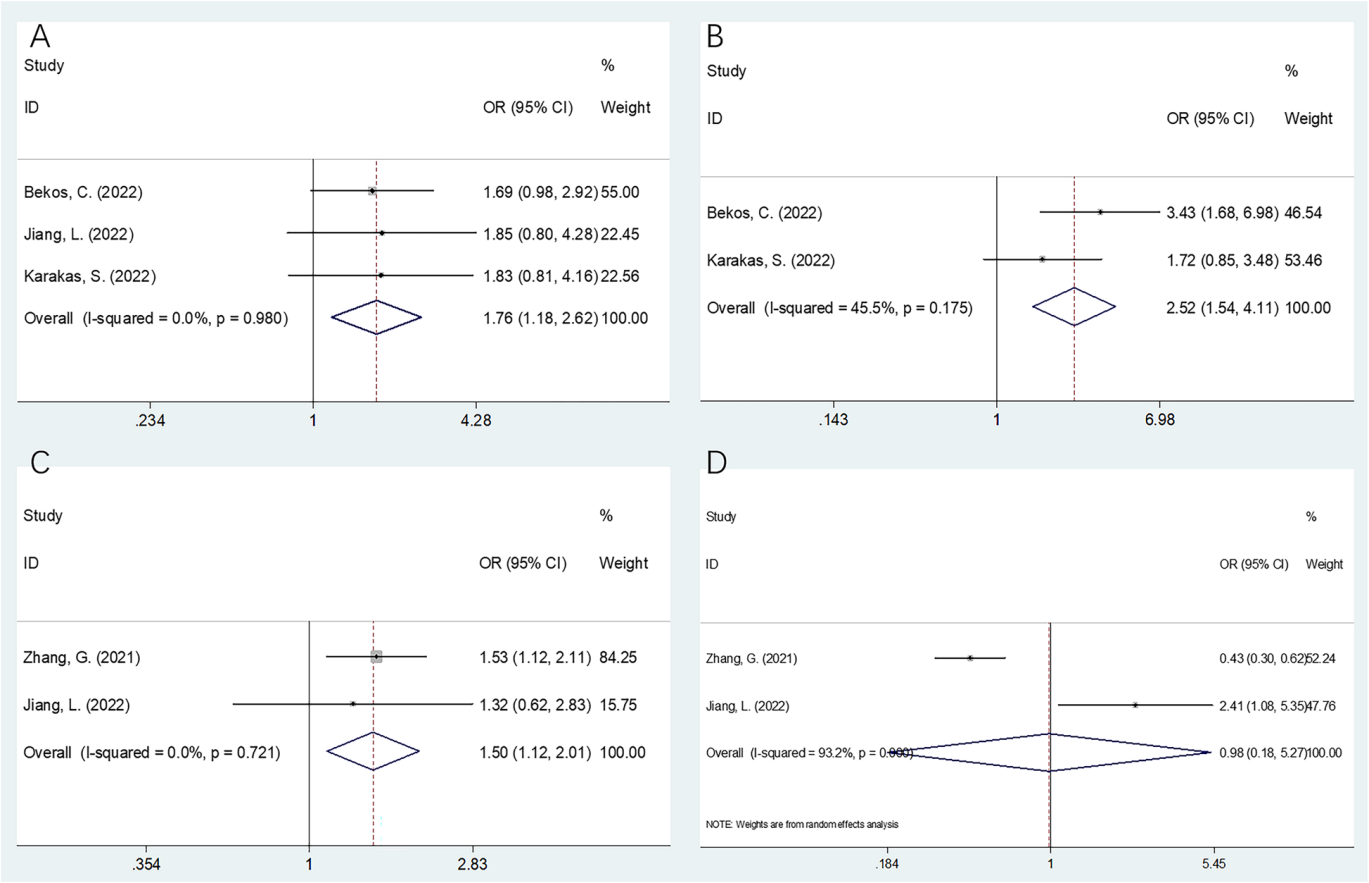
The correlation between CONUT and clinicopathological factors in gynecological cancer. **A** Histological grade (G3 vs G1-G2); **B** FIGO stage (III-IV vs I-II); **C** Tumor size (≥ 4 cm vs < 4 cm); and **D** Lymph node metastasis (presence vs absence)

Of the articles included for analysis in the present study, a total of 4 studies, involving 1,325 patients with gynecological cancer [13–16], reported a relationship between the CONUT score and clinicopathological factors of gynecological cancer. As shown by the combined results in Fig. 4 and Table 5, increased CONUT scores were significantly associated with a histological grade of G3 (OR = 1.76; 95% CI = 1.18–2.62; *P* = 0.006), a tumor size ≥ 4 cm (OR = 1.50; 95% CI = 1.12–2.01; *P* = 0.007), and an advanced FIGO stage (OR = 2.52; 95% CI = 1.54–4.11; *P* < 0.001). There was no significant correlation, however, between the CONUT score and lymph node metastasis (OR = 0.98; 95% CI = 0.18–5.27; *P* = 0.984) (Fig. 4; Table 5).

**Table 5:** The correlation between CONUT and clinicopathological characteristics in patients with gynecological cancer

Variables	No. of studies	No. of patients	Effects model	OR (95% CI)	p	Heterogeneity	
						I^2^(%)	Ph
Histological grade (G3 vs G1-G2)	3	627	Fixed	1.76 (1.18–2.62)	0.006	0	0.980
FIGO stage (III-IV vs I-II)	2	505	Fixed	2.52 (1.54–4.11)	< 0.001	45.5	0.175
Tumor size (≥ 4 cm vs < 4 cm)	2	820	Fixed	1.50 (1.12–2.01)	0.007	0	0.721
Lymph node metastasis (presence vs absence)	2	820	Random	0.98 (0.18–5.27)	0.984	93.2	< 0.001

*CONUT* controlled nutritional status score, *FIGO* International Federation of Gynecology and Obstetrics

### Sensitivity analysis

**Fig. 5:**
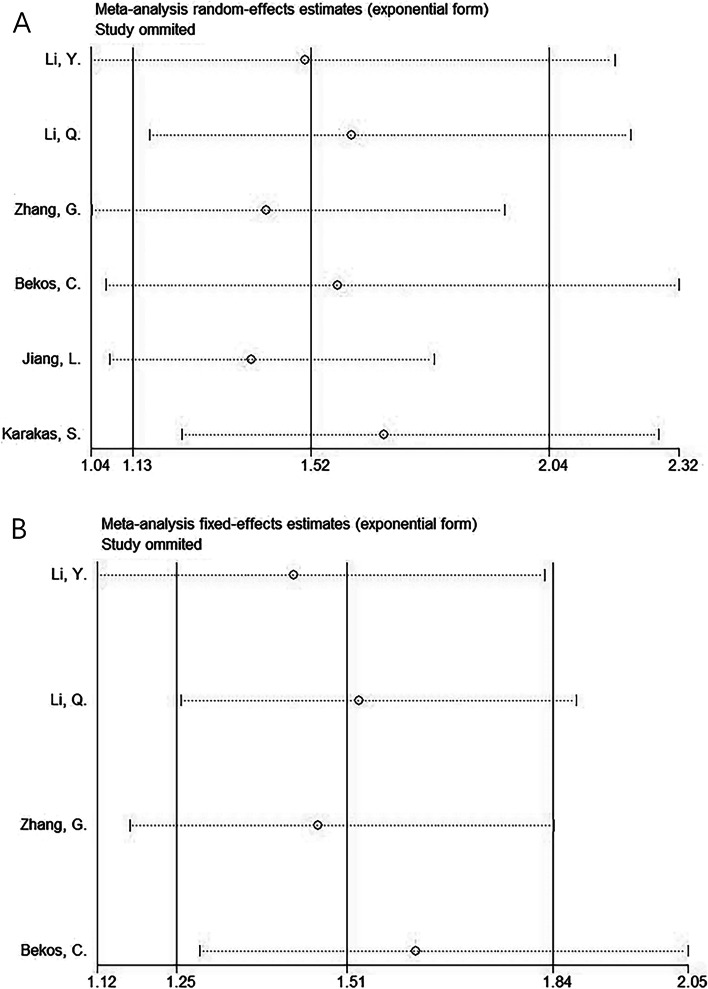
Sensitivity analysis. **A** OS; and **B** PFS

In the present study, we performed a sensitivity analysis on the relationship between the CONUT score and OS and PFS (Fig. 5), through which we determined that the significance of the CONUT score in predicting OS and PFS in patients with gynecological cancer did not change after eliminating any single article (Fig. 5). The detailed results for OS and PFS are shown in Supplementary file 1.

### Publication bias

**Fig. 6:**
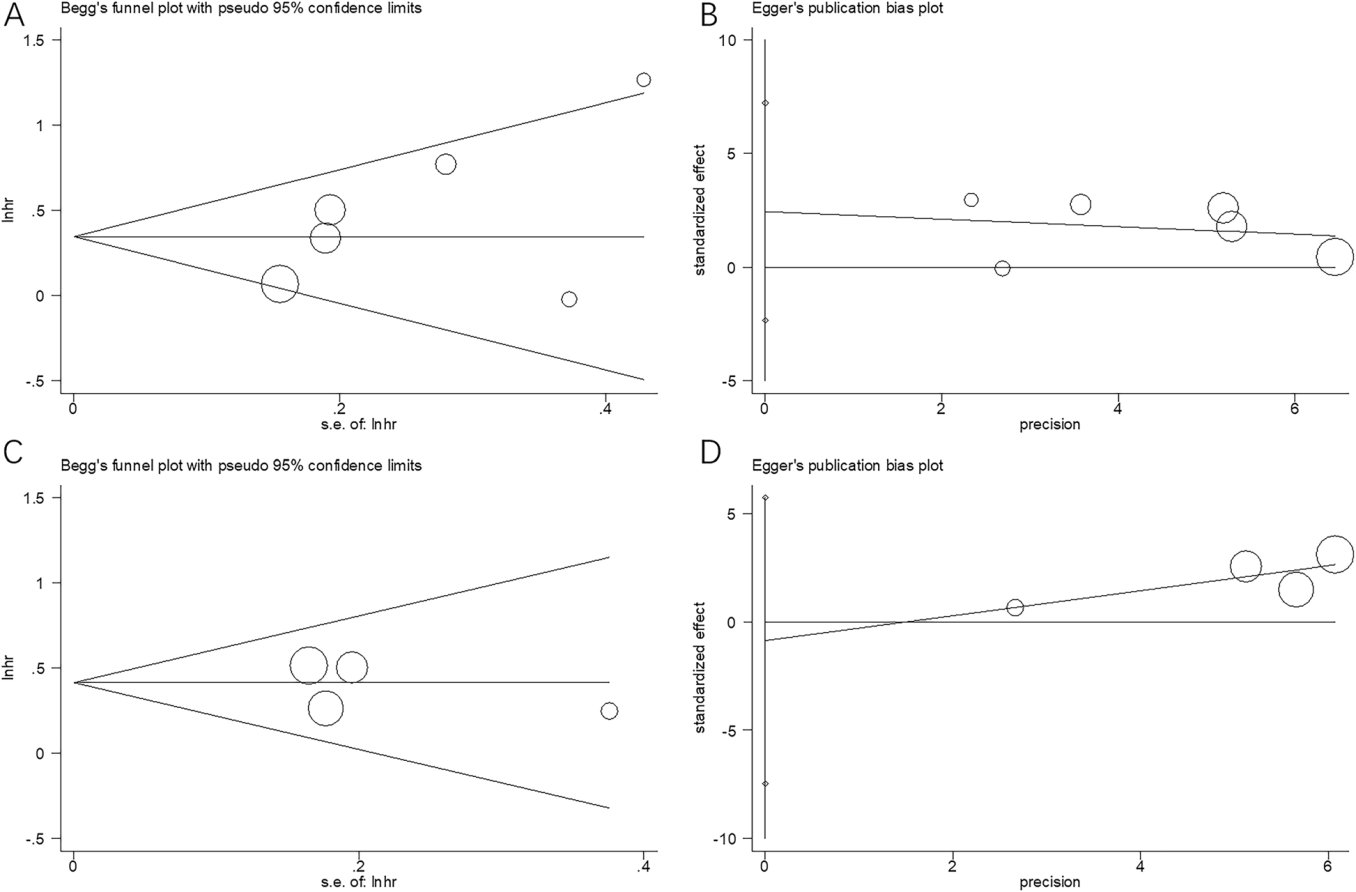
Publication bias in this meta-analysis. **A** Begg’s test for OS, *p* = 0.133; **B** Egger’s test for OS: *p* = 0.229; **C** Begg’s test for PFS: *p* = 0.734; and **D** Egger’s test for PFS: *p* = 0.631

A funnel plot was constructed to analyze the significance of the CONUT score in predicting OS (Fig. 6A) and PFS (Fig. 6C) in patients with gynecological cancer. No asymmetry was observed in the funnel plot, suggesting no evidence of publication bias. Moreover, according to Egger’s (*P* = 0.229 and *P* = 0.631) and Begg’s (*P* = 0.133 and *P* = 0.734) tests for OS and PFS, respectively, publication bias was not observed in the present meta-analysis (Fig. 6).

## Discussion

The significance of the CONUT score in predicting the outcomes of patients with gynecological cancer has been controversial according to the results of previous studies [10, 12–16]. In the present meta-analysis, we collected information from 6 articles, encompassing 2,569 cases of gynecological cancer, and then systematically analyzed the relationship of the CONUT score with OS and PFS in patients with gynecological cancer. According to the results of the present meta-analysis, increased CONUT scores were significantly correlated with poor OS and PFS in patients with gynecological cancer. Furthermore, the prognostic significance of the CONUT score remained stable in its correlation to OS and PFS, regardless of the FIGO stage or study center. Additionally, according to the results of the present meta-analysis, increased CONUT scores were significantly associated with an advanced FIGO stage, poor tumor differentiation, and an increased tumor size, corresponding to highly malignant tumors. Therefore, patients with gynecological cancer who have higher CONUT scores may be at an increased risk of tumor progression and have poorer prognoses. The clinical management of these patients should be intentional, and more aggressive treatment regimens should be considered. To the best of our knowledge, the present study is the first to investigate the prognostic value of the CONUT score in patients with gynecological cancer.

The CONUT score consists of three elements: serum albumin (ALB), lymphocytes, and cholesterol (Table 1). Therefore, the potential mechanisms of the CONUT score as a prognostic marker for gynecological cancer are interpreted as follows. First, serum ALB is a vital biomarker of nutritional status. Pretreatment hypoalbuminemia indicates a state of malnutrition and is usually secondary to cancer in patients, especially those at an advanced stage [20, 21]. Second, lymphocytes have important effects on the anticancer activity of the immune system, as they can prevent tumor cell growth by enhancing cytotoxic cell apoptosis and suppressing cancer cell growth and invasion [22]. Decreased levels of tumor-infiltrating lymphocytes (TILs) indicate worse survival odds in patients with cancer [23]. Third, cholesterol protects against signal transduction and maintains cell membrane fluidity, activity, and integrity [24]. Additionally, cholesterol can promote the development of antigen-presenting monocytes, enhancing the antitumor activity of TILs in the tumor microenvironment. As a result, increased CONUT scores, which are the result of decreased serum ALB, lymphocytes, and cholesterol levels, are a reasonable indicator of poor survival in patients with gynecological cancer. According to the findings of the present study, an increased CONUT score can predict an advanced FIGO stage, poor tumor differentiation, and a large tumor size. These results suggest that gynecological cancer patients with higher CONUT scores often experience more aggressive and rapidly growing malignancies.

Of note, significant heterogeneity was detected in the prognostic value of the CONUT score for OS (Table 3). A subgroup analysis was performed to determine the source of heterogeneity, which revealed that studies conducted in China, those with a sample size ≥ 300, those involving OC and CC, those involving patients treated with surgery + CCRT, and those with a CONUT threshold score of 3 showed consistent prognostic efficiency. These results suggest that studies with a sample size ≥ 300 and a CONUT cutoff value of 3 tended to identify the prognostic value of CONUT. No evidence of heterogeneity was found in the analysis of the prognostic value of the CONUT score for PFS (Table 4).

Numerous recent studies have analyzed the significance of the CONUT score in a variety of cancer types [25–28]. For example, based on a recent meta-analysis enrolling 1,811 cases, an increased CONUT score predicted poor OS and PFS in hematologic cancer cases and, therefore, independently predicted patient prognosis [25]. Ma et al. performed a meta-analysis of seven studies, and according to their results, an increased CONUT score predicted poor OS in patients with pancreatic cancer [27]. Peng et al. showed that an increased CONUT score indicated worse OS, disease-free survival (DFS), recurrence-free survival (RFS), and cancer-specific survival (CSS) in patients with upper urinary tract urothelial and renal cell carcinoma, based on a meta-analysis involving 5,410 cases [28]. According to another recent meta-analysis, higher CONUT scores predicted poorer OS, DFS, CSS, and PFS compared to lower CONUT scores in patients with lung cancer [29]. As suggested by the results of a meta-analysis of 2,601 cases by Takagi et al., an increased CONUT score predicted poor OS, CSS, and RFS in patients with colorectal cancer undergoing surgical resection [30].

The present meta-analysis does have some limitations, which should be noted. First, this meta-analysis had a small sample size and included only six articles; therefore, the sample size should be expanded in future studies. Second, each of the enrolled articles followed a retrospective design; therefore, an inherent selection bias could not be avoided, which may have affected the quality of the evidence. Third, there was not a uniform CONUT score threshold among the articles included in the present meta-analysis; therefore, subgroup analysis of the cutoff values was performed and indicated that a CONUT score cutoff of 3 showed reliable prognostic value (Tables 3 and 4). Each of the aforementioned factors may have contributed to the heterogeneity of the present meta-analysis. As a standard and uniform CONUT threshold is needed, large-scale prospective clinical trials are needed to validate the findings of the present meta-analysis.

## Conclusions

In conclusion, increased CONUT scores were significantly associated with poor OS and PFS in patients with gynecological cancer. Moreover, increased CONUT scores showed a significant relationship with an advanced FIGO stage, poor tumor differentiation, and a large tumor size in gynecological cancer patients. The results of the present study, therefore, indicate that the CONUT score can serve as a promising and cost-effective biomarker for the prognostication of gynecological cancer patients.

## Supplementary Information

**Additional file 1:** **Supplementary file Table S1.** Results of sensitivity analysis for OS and PFS.

**Additional file 2:** **Supplementary file 2.** The Editing Certificate from American Journal Experts (https://www.aje.com/).

## Data Availability

The data that support the findings of this study are available from the corresponding author upon reasonable request.

## Abbreviations

CONUT: Controlling nutritional status
HR: Hazard ratio
CI: Confidence interval
CC: Cervical cancer
OC: Ovarian cancer
EC: Endometrial cancer
PRISMA: Preferred Reporting Items for Systematic Reviews and Meta-Analyses
NOS: Newcastle-Ottawa Scale
OR: Odds ratio
FIGO: International Federation of Gynecology and Obstetrics
OS: Overall survival
PFS: Progression-free survival
TIL: Tumor-infiltrating lymphocyte
ALB: Albumin
CSS: Cancer-specific survival
DFS: Disease-free survival
RFS: Recurrence-free survival
TC: Total cholesterol
NLR: Neutrophil to lymphocyte ratio
NPS: Naples prognostic score
BMI: Body mass index
SCC-Ag: Squamous cell carcinoma antigen
PNI: Prognostic nutritional index
NPS: Naples prognostic score
SCC-Ag: Squamous cell carcinoma antigen
