# Supplementary material for: Prognostic and clinicopathological impacts of Controlling Nutritional Status (CONUT) score on patients with gynecological cancer: a meta-analysis
Source: Nutr J. 2023 Jul 8;22:33. doi: 10.1186/s12937-023-00863-8 (PMC10329389; doi:10.1186/s12937-023-00863-8)
Supplement: Supplementary file 1 — Additional file 1: Supplementary file Table S1. Results of sensitivity analysis for OS and PFS. [file 12937_2023_863_MOESM1_ESM.docx]

Supplementary file Table S1 Results of sensitivity analysis for OS and PFS.

| Study excluded | HR (95% CI) | p |
| --- | --- | --- |
| OS |  |  |
| Li, Y. (2020) | 1.50(1.04-2.18) | 0.004 |
| Li, Q. (2021) | 1.61(1.16-2.21) | <0.001 |
| Zhang, G. (2021) | 1.42(1.04-1.94) | 0.002 |
| Bekos, C. (2022) | 1.58(1.07-2.32) | <0.001 |
| Jiang, L. (2022) | 1.39(1.08-1.79) | <0.001 |
| Karakaş, S. (2022) | 1.68(1.23-2.28) | <0.001 |
| PFS |  |  |
| Li, Y. (2020) | 1.43(1.12-1.82) | <0.001 |
| Li, Q. (2021) | 1.53(1.25-1.87) | <0.001 |
| Zhang, G. (2021) | 1.47(1.17-1.84) | 0.002 |
| Bekos, C. (2022) | 1.62(1.28-2.05) | <0.001 |
